# A Case Report and Literature Review of Steroid-Resistant Granulomatous Mastitis: Dramatic Response to Methotrexate Following Failed Drainage and Corticosteroid Therapy

**DOI:** 10.7759/cureus.101219

**Published:** 2026-01-10

**Authors:** Feras Buhusayen, Amena F Almubarak, Mooza Alabbasi, Kaltham Bedaiwi, Noora Almoosa

**Affiliations:** 1 General Surgery, Bahrain Defence Force Hospital, Royal Medical Services, Riffa, BHR; 2 General Surgery, King Hamad University Hospital, Al Sayh, BHR; 3 General Practice, Bahrain Defence Force Hospital, Royal Medical Services, Riffa, BHR; 4 Breast and Endocrine Surgery, Bahrain Defence Force Hospital, Royal Medical Services, Riffa, BHR

**Keywords:** breast inflammatory disease, idiopathic granulomatous mastitis (igm), immunosuppressive treatment, methotrexate therapy, steroid-resistant granulomatous mastitis

## Abstract

Idiopathic granulomatous mastitis is an uncommon benign inflammatory breast condition that often follows a prolonged and relapsing course and may closely resemble infection or malignancy, leading to repeated interventions. We describe a young multiparous woman with a long history of recurrent breast abscesses and persistent draining sinuses who failed multiple surgical drainages and prolonged systemic corticosteroid therapy. Ongoing disease activity prompted a shift in management to immunomodulatory treatment, after which the patient experienced gradual but complete clinical and radiologic resolution with sustained remission on follow-up. This case illustrates the potential role of methotrexate as an effective alternative in refractory disease and highlights the importance of considering steroid-sparing strategies to reduce morbidity and avoid unnecessary repeated surgical procedures.

## Introduction

Idiopathic granulomatous mastitis (IGM) is a rare, benign, chronic inflammatory disease of the breast with an uncertain etiology [1]. The disease typically develops gradually over weeks to months and frequently mimics both infectious mastitis and breast malignancy, creating significant diagnostic uncertainty. Although symptoms may resolve spontaneously over time, many patients experience a protracted course marked by recurrent pain, abscess formation, and sinus tract development [1]. It typically affects women of childbearing age, often presenting postpartum. Clinically and radiologically, IGM can closely mimic breast infections or even malignancy [1]. This condition often necessitates an exhaustive workup to exclude common infectious causes (such as Mycobacterium or Corynebacterium species) and breast cancer before arriving at the diagnosis of IGM [2]. IGM is characterized histologically by sterile, noncaseating granulomatous inflammation [1]. The condition was first recognized in the 1970s, yet its pathogenesis remains poorly understood, and it continues to pose a diagnostic and clinical challenge given its unpredictable, relapsing course [2].

Management of IGM is equally challenging, as there is no single established treatment protocol and multiple modalities have been employed [3]. Conventional approaches include broad-spectrum antibiotics (often empirically given prior to confirming the sterile granulomatous nature of the disease), corticosteroid therapy (local intralesional injection or systemic), and surgical intervention (ranging from abscess drainage to wide excision and mastectomy) [3]. Each of these strategies has limitations. Antibiotics do not alter the course of IGM since the inflammation is noninfectious. Furthermore, steroids can induce remission, but prolonged courses are frequently required, with high relapse rates upon tapering and significant side effects from long-term use. Surgery can be disfiguring or incomplete, with recurrences reported even after mastectomy or repeated drainage [4].

In recent years, steroid-sparing immunosuppressants such as methotrexate (MTX) have emerged as promising alternatives for refractory or recurrent IGM [3]. MTX, alone or combined with low-dose prednisone, has been shown to induce remission in patients who fail first-line therapies, while also reducing relapse rates and allowing tapering of corticosteroids [4]. Given the rarity of IGM and the paucity of definitive guidelines, we present a case of IGM unresponsive to conventional corticosteroids and surgical drainage that was successfully treated with MTX. This case underscores the potential role of MTX as a therapeutic option in steroid-resistant IGM and highlights the importance of considering alternative immunosuppressive therapy in managing this uncommon and challenging breast inflammatory condition.

## Case presentation

A 35-year-old woman, who is married and has five children, has no significant medical history and does not have any history of allergies, smoking, or alcohol consumption. She presented with a four-year history of recurrent right breast abscesses attributed to granulomatous mastitis of the right breast. She reported intermittent swelling, persistent pain, and foul-smelling seropurulent discharge associated with overlying erythema. Despite multiple incision and drainage (I&D) procedures and prolonged courses of systemic corticosteroids (40 mg for one month, then 35 mg for one month, then 30 mg for one month, then 25 mg for 10 months), her symptoms persisted, and new collections continued to develop. No systemic symptoms were reported, though she noted fatigue and weakness. On examination, the patient was afebrile and clinically stable. Local examination revealed a tender lower quadrant breast mass with multiple discharging sinus tracts and surrounding inflammatory changes.

This case was first presented in December 2022 at the Royal Medical Services - Military Hospital in Riffa, Bahrain. The patient initially presented to the breast clinic in December 2022 with recurrent right breast abscesses, later requiring admission for surgical evaluation and management. She continued to receive follow-up care through 2023-2025 at the same facility, with repeated clinical reviews, ultrasound assessments, I&D procedures, and adjustments to medical therapy as her condition progressed.

**Figure 1:**
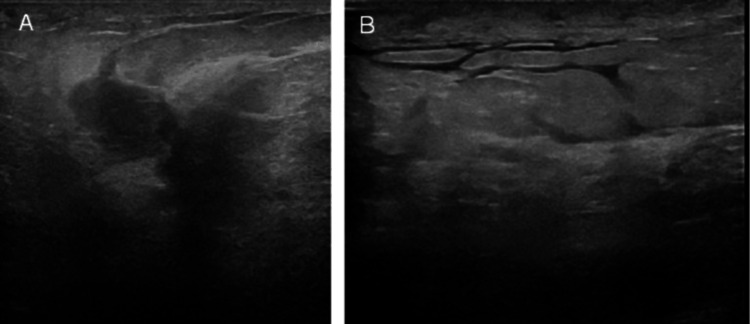
(A) Ill-defined hypoechoic areas with fingerlike projections. (B) Cobblestone appearance indicating tissue edema and overlying skin thickening

All laboratory values were within normal limits. Right breast ultrasound showed multiple collections, the largest noted at 8 o'clock measuring 2 x 3 cm, and another collection at 9 o'clock measuring 3.7 x 3 cm with other smaller collections scattered through the breast (Figure 1). Ultrasound of the right axilla demonstrated a right axillary inflammatory lymph node with no hilum measuring 1.5 x 0.7 cm. There were also retroareolar collections measuring 1.4 x 2 cm.

Multiple tissue samples obtained between December 2022 and June 2024 confirmed the diagnosis of granulomatous mastitis. The earliest specimen (December 2022) demonstrated cystic neutrophilic granulomatous mastitis with abscess formation, acute inflammatory infiltrates, epithelioid histiocytes, and multinucleated giant cells, with no evidence of ductal carcinoma in situ or invasive malignancy; special stains for AFB, fungi, and other infectious pathogens were negative. Subsequent excision in May 2023 again showed cystic neutrophilic granulomatous mastitis with chronic inflammatory changes and cystic areas, along with negative fungal and acid-fast bacilli stains, further supporting a noninfectious inflammatory etiology. A tru-cut biopsy performed in June 2024 reconfirmed granulomatous mastitis without in situ or invasive carcinoma, and supplementary stains again excluded infectious causes, consolidating the diagnosis and guiding the shift toward immunomodulatory therapy.

The patient was admitted for persistent pain and discharge. I&D was performed and revealed multiple pus cavities. Postoperative course was uneventful; a vacuum-assisted closure dressing was applied with twice-weekly changes and discharged after two days in stable condition with analgesics, proton pump inhibitor, and close follow-up in the breast clinic.

**Figure 2:**
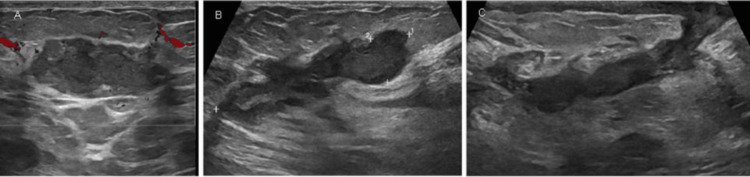
(A,B) Multiple turbid fluid collections. (C) Sinus track opening at the skin

**Figure 3:**
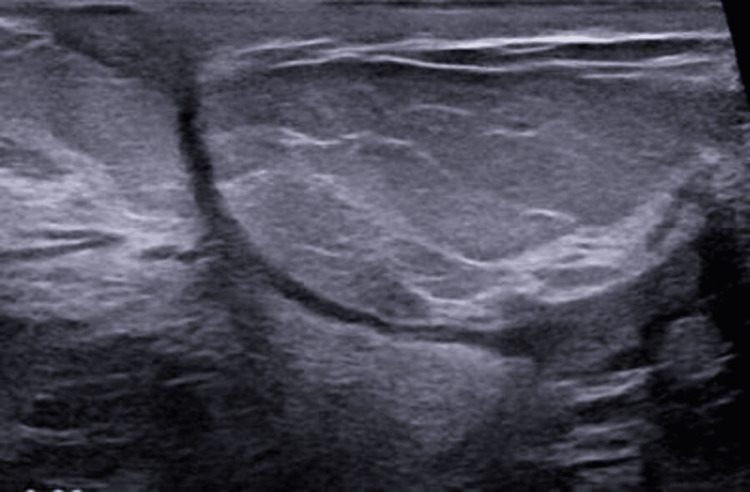
After treatment initiation sinus started to close and breast changes were improving

**Figure 4:**
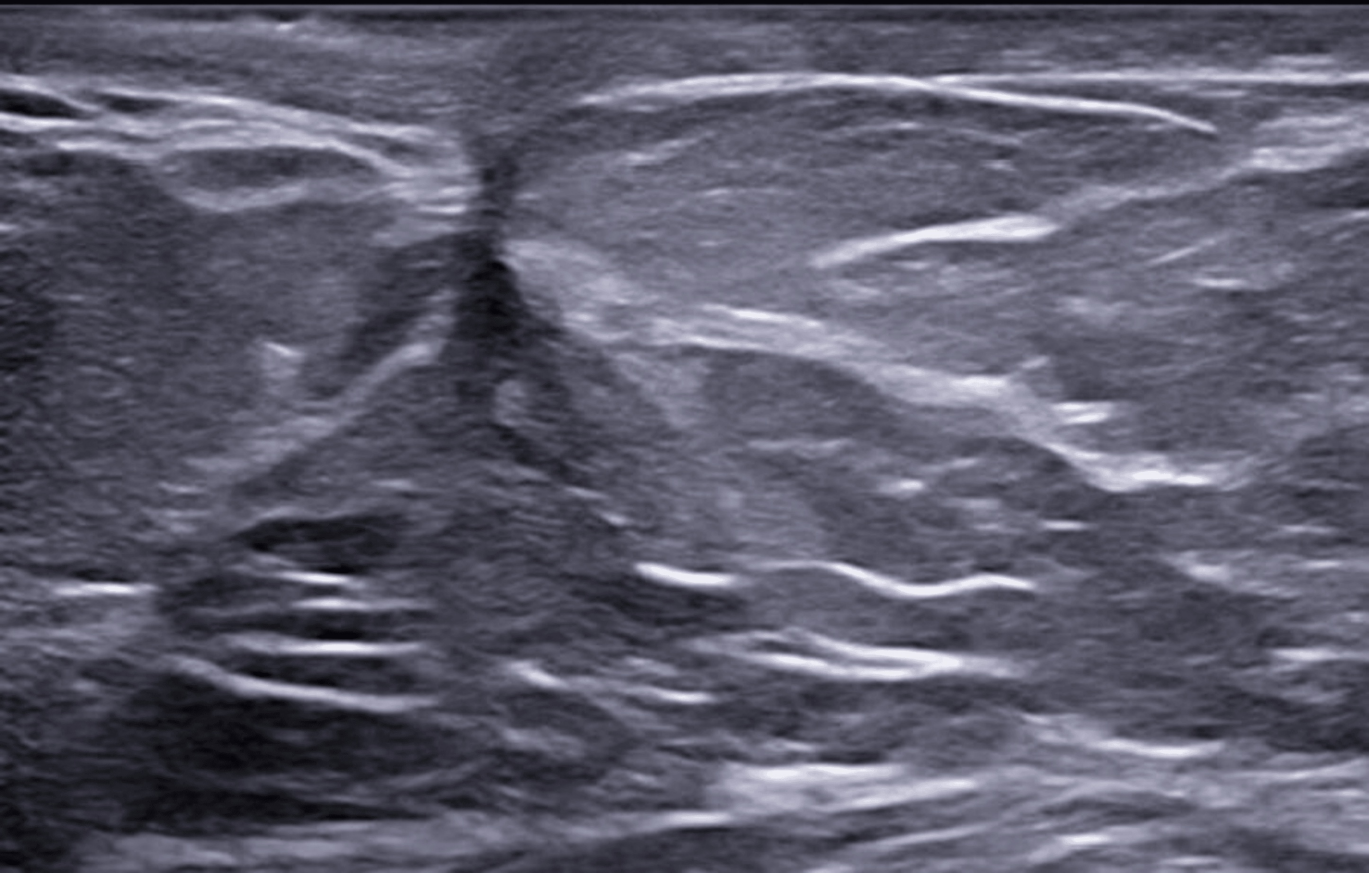
Post-treatment scar tissue at the site of the previous sinus with normal surrounding breast parenchyma

Despite multiple courses of oral prednisolone and three attempts of I&D, the patient continued to experience intermittent breast pain, pricking, and tenderness in addition to ongoing seropurulent discharge. Examination showed redness and localized swelling. Post-I&D ultrasound demonstrated persistent heterogeneous echotexture and new collections, suggesting reactivation rather than postsurgical change (Figure 2). The patient started on MTX 7.5 mg weekly and folic acid following a multidisciplinary discussion with rheumatology (all pros and cons were discussed). Over the subsequent months, the patient reported marked clinical improvement, with gradual resolution of her symptoms and complete healing of previously discharging wounds. On examination, the breast became soft and nontender with no palpable masses, erythema, or discharge. Follow-up ultrasounds demonstrated significant regression of inflammatory changes, showing only postoperative fibrosis and a small simple cyst without any abscess or fluid collection (Figure 3). The patient tolerated MTX well, with no adverse effects, and remained asymptomatic throughout 2024-2025. Sustained remission was confirmed radiologically (Figure 4) and clinically after more than one year of therapy.

## Discussion

IGM is a benign, chronic inflammatory breast disease that often mimics infection or malignancy and presents a diagnostic and therapeutic challenge. Although corticosteroids have long been considered first-line therapy, relapses and steroid-dependency are frequent, leading to exploration of immunosuppressive alternatives such as MTX. Over the past decade, accumulating evidence from both case-based experiences and larger observational studies has positioned MTX as the most effective and durable medical option for steroid-refractory IGM.

Literature review methodology

To contextualize this case and evaluate current evidence regarding the use of MTX in IGM, a focused literature review was conducted. Electronic databases, including PubMed, Scopus, and Google Scholar, were searched for English-language articles published using the terms “idiopathic granulomatous mastitis”, “methotrexate”, “steroid-resistant”, and “treatment”. Case reports, case series, and observational studies that specifically discussed MTX therapy for IGM were included. Articles reporting non-IGM or treatment modalities unrelated to MTX were excluded. Relevant data regarding patient characteristics, treatment protocols, clinical outcomes, and follow-up duration were extracted and synthesized to provide a comprehensive overview of current therapeutic evidence.

Evidence from case reports

Early insights into MTX efficacy were derived from multiple case reports and small series between 2009 and 2025 (Table 1). These reports consistently documented remission in patients who had failed corticosteroids, drainage, or prolonged antibiotics. MTX doses generally ranged between 7.5 and 20 mg weekly, administered either alone or with low-dose prednisone. The time to clinical remission varied from three to six months, and most patients remained in long-term remission on maintenance therapy.

**Table 1:** All identified case reports of MTX treated granulomatous mastitis EN: erythema nodosum; ER+: estrogen receptor positive; PR-: progesterone receptor negative; GM: granulomatous mastitis; GMENA: granulomatous mastitis erythema nodosum and arthritis; GPA: granulomatosis with polyangiitis; IGM: idiopathic granulomatous mastitis; MTX: methotrexate

Study	Country/type	Patient(s)	Systemic or associated findings	MTX regimen	Steroid use	Outcome/follow-up	Key notes
Ribeiro et al. [5]	Portugal/Case report	39 F, pregnant with twins	Erythema nodosum, left-breast IGM	5-10 mg/week (oral)	Prednisolone 30 mg/day	Complete remission on MTX; maintained on therapy	IGM with EN in pregnancy; excellent MTX response
Akbari Rad et al. [6]	Iran/Case + Review	46 F, premenopausal	Coexistent invasive ductal carcinoma (ER+/PR-)	12.5-15 mg/week	Prednisolone (initial, 9 months)	Complete remission of IGM; cancer managed surgically	First IGM-breast cancer co-occurrence; MTX effective, no recurrence
Abuhammad et al. [11]	Palestine-USA/Case + Review	31 F	Sarcoid-like GM with arthritis + erythema nodosum (GMENA)	20 mg/week	Prednisolone 20 mg/day (tapered)	Full remission in 6 months; maintained on low-dose MTX	GM as sarcoidosis variant; MTX + steroid curative
Bothara et al. [12]	USA/Case report	37 F	GPA presenting as recurrent breast abscesses	15 mg/week	Prednisolone 20 mg/day → tapered off	Complete remission; no recurrence	GPA initially misdiagnosed as recurrent infection; MTX curative
Lu et al. [13]	Taiwan/Case + Review	33 F, androgynous	None; sulpiride exposure (hyperprolactinemia risk)	7.5 mg/week (with steroid)	Prednisolone 30 mg BID	Failed MTX + steroid; cured after double mastectomy	First report of “top surgery” cure after MTX failure
Hashmi et al. [14]	UK/Case report	26 F, Caucasian	Erythema nodosum	10 mg/week (oral)	Prednisolone 20 mg/day → refractory	Complete radiologic and clinical remission after 6 month MTX	Steroid-refractory IGM + EN; sustained remission for 30 months
Wankhedkar et al. [15]	USA/Case report	33 F, Hispanic, recurrent bilateral mastitis	None reported; recurrent abscesses misdiagnosed as infection	10-20 mg/week (dose escalated)	Prednisone added during nodularity; tapered later	Complete remission; disease-free at follow-up; no adverse effects	Refractory to antibiotics and I&D; biopsy confirmed IGM; MTX ± steroids effective and well tolerated
Nakamura et al. [16]	Japan/Case report	23 F	Erythema nodosum + polyarthritis	4 mg/week	Prednisolone 40 mg/day (relapse during taper)	Full remission; maintained on low-dose steroid; relapse-free 2 years	Classic IGM + EN + arthritis; MTX effective steroid-sparing agent
Schmajuk and Genovese [17]	USA/Case series (2 pts)	32 F and 35 F, Asian/South Asian	Mild arthralgia in 1	15-20 mg/week (monotherapy)	None	Complete remission in both (12-24 months)	First report of MTX monotherapy; steroid-free durable remission

Several recurring clinical associations, including erythema nodosum, polyarthritis, pregnancy, and hyperprolactinemia, were identified, suggesting an autoimmune or hormone-modulated component to the disease. Notably, Ribeiro et al. described a pregnant patient with erythema nodosum who achieved full remission on MTX [5], whereas Akbari Rad et al. reported successful remission even in a case complicated by coexistent breast carcinoma [6]. Across all identified reports, nearly all patients achieved remission, and relapses were uncommon, typically following premature discontinuation of therapy. Collectively, these individual experiences established the clinical rationale for incorporating MTX into standardized treatment algorithms.

Evidence from observational studies

The findings from recent observational studies further reinforce the role of MTX as an effective and reliable therapeutic option in the management of IGM, particularly in cases refractory to corticosteroids or surgical drainage (Table 2). Across multiple retrospective analyses conducted between 1990 and 2025 in diverse settings, including Turkey, Canada, and the United States, MTX consistently demonstrated high remission rates and sustained disease control. The largest study by Kaya et al. included 114 biopsy-proven IGM patients treated with MTX monotherapy for at least one year, achieving complete remission in 85.1% of cases, with a mean treatment duration of approximately 11 months [7]. Similarly, Mourot et al. applied a disease-modifying antirheumatic drug-based approach in 22 patients and reported 95% complete remission, with only a single relapse observed during follow-up [8]. A separate Turkish series by Kaya et al. confirmed durable remission and the absence of recurrence after a five-year follow-up among patients who received immunosuppressive therapy, primarily MTX [9]. In the U.S. cohort by Abbi et al., MTX was part of combination regimens, and despite a smaller sample size (7/27 patients on MTX), the response remained favorable, with relapse occurring in 29% of MTX users, likely reflecting variability in treatment duration or concurrent hormonal influences, as oral contraceptive use was linked to recurrence [10].

**Table 2:** All identified observational studies of MTX-treated granulomatous mastitis ABS: esthetic breast surgery; AEs: adverse events; AZA: azathioprine; CR: case report; DMARD: disease modifying anti-rheumatic drug; ESR: erythrocyte sedimentation rate; CRP: C-reactive protein; FU: follow-up; GLM: granulomatous lobular mastitis; IS: immunosuppressive; LFT: liver function test; MTX: methotrexate; NYC: New York City; OCP: oral contraceptive pill

Study	Country/setting	Design/period	n (total)	n on MTX	MTX regimen (mono/combination)	Key outcomes (remission/recurrence)	Follow-up/durations	Notes
Kaya et al. [7]	Turkey	Retrospective; 2017-2024	114	114	Monotherapy, ≥1 year	CR 85.1% (97/114); KM mean MTX use 11.24 months; “well tolerated”	95% CI for use 10.88-11.49 months	-
Mourot et al. [8]	Canada	Retrospective case series	22	9	DMARD-based algorithm; MTX most used	CR 95% overall; 1 relapse (~4.5%); mean time to remission 11.6 months	Mean rheum FU 28.7 months	Corynebacterium in 8; severity-based approach
Kaya et al. [9]	Turkey	Retrospective; 5-year follow-up	63	NR (MTX most preferred)	Immunosuppressants (MTX favored)	All on IS achieved remission; 0 recurrences over 5 years	Median remission 13.9 months; drug-free remission 46.1 months	Agent-level split not provided
Abbi et al. [10]	USA (Montefiore)	Retrospective; 1990-2021	27	7	Usually combo; start 15 mg/week	In MTX group: relapse 29% (2/7); cohort CR 67%; relapse 33% overall	-	OCP use linked to relapse; no surgery used
Esmaeil et al. [18]	Iraq (Sulaimani)	Single-group cohort; 2020-2022	63	10	Combo MTX 7.5 mg tabs + low-dose steroids	CR 30%, 70% partial/recurrence in MTX group	Mean time to resolution 16.2 months	In this cohort, I&D + low-dose steroids outperformed MTX+steroid
Kaya et al. [19]	Turkey	Retrospective cohort; 3-year outcomes	55	NR	CS ± IS; MTX subgroup analyzed	Drug-free remission 98.1% by 3 years; MTX DFR 19.7 months; CS only 32.9 months	Med IS duration 15.8 months; CS+IS 6.7 months	4 relapses after surgery alone; 3 responded to MTX
Tian et al. [20]	China (Chengdu)	Retrospective; 2015-2022	81 (steroid-resistant GLM)	81	Combo MTX 7.5 mg/week + low-dose methylpred	Overall response 75.3% (CR 54.3%); relapse 6 pts; MTX-resistance 23.5%	Median FU 18 months; Tx mean 9 months	Mild AEs: 1 LFT ↑ (1.2%), hair loss 6.2%
Papila Kundaktepe et al. [21]	Turkey (Istanbul Univ.)	Retrospective case series; 2013-2020	64	64	Monotherapy 15→20 mg/week; ~24-52 week	CR 81.3%; relapse 12.5%	Tx ≈ 265 ± 126 days; FU ≈ 780 days	Largest MTX-mono series; dose increase controlled relapses; good tolerance
Dalbaşı and Akgül [22]	Turkey (Diyarbakır)	Retrospective; 2009-2017	62	62	Combo MTX 15→25 mg/week + low-dose methylpred	Recovery 93.7%; relapse 11.3%	Mean Tx ~14 months; FU 18-36 months	No discontinuations; significant ESR/CRP/mass reductions
Ringsted and Friedman [23]	USA (OHSU)	Case series; 2007-2018	28 (local)	5 (local)	MTX ± steroids 15-20 mg/week	Local MTX: 80% remission; 0% relapse; Review (116 MTX pts): ~79% remission	FU 13-34 months (MTX pts)	MTX highest relapse-free remission vs. steroids alone
Kafadar et al. [24]	Turkey (Dicle Univ.)	Retrospective; 2010-2016	17	17	Combo MTX 5 mg/week + prednisone 8 mg/day	CR 58.8%, PR 17.6%, NR 23.5%; 0% recurrence	FU 8-9 months	Very low-dose MTX; no AEs reported
Berkeşoğlu et al. [25]	Turkey (postesthetic surgery subset)	Retrospective; 2010-2019	6 GM post-ABS	2	Weekly MTX 10-15 mg + steroids/surgery as needed	No recurrence in both MTX cases	FU 18-70 months	Subgroup: postreduction/augmentation GM; high smoking rate
Kehribar et al. [26]	Turkey	Retrospective cohort; 2013-2016	33	33	Combo MTX 7.5-15 mg/week + methylpred	CR 87.9%; 0% recurrence (24 months)	Mean Tx 12.8 months; FU 24 months	Many maintained remission on MTX mono after steroid taper
Postolova et al. [27]	USA (Stanford)	Retrospective case series	19	19	Monotherapy 10-25 mg/week (PO→SC if needed)	Improve 94%; CR 75%; relapse on-tx 15.8% (managed)	Median Tx 13-15 months; FU ~3 years	One failure → mastectomy; mild reversible AEs
Tekgöz et al. [28]	Turkey (Gülhane)	Retrospective; 2011-2018	53	41	Combo MTX (med. 15 mg/week) + steroids	CR 80.5% (MTX grp); relapse 12.2%	Med. Tx 9.1 months; time to remission ~13 months	Rapid clinical improvement in 1 month; switch to AZA if intolerant
Haddad et al. [29]	Iran (Mashhad)	Retrospective; -	17	13	Mono (n = 7)/Combo (n = 6), 7.5-15 mg/week	100% remission in MTX-treated; relapse 17.6% (all re-responded to MTX)	Mean FU 16.4 months	First-line MTX strategy evaluated; mild AEs
Freeman et al. [30]	USA (Cincinnati)	Retrospective; 2004-2016	14	2	Second-line MTX (dose NR)	1 improved; 1 intolerant → mastectomy; cohort relapse 14%	-	Small US experience; mixed outcomes
Sheybani et al. [31]	Iran (Mashhad)	Prospective cohort	22 idiopathic (of 28)	7 mono + 6 combo (12 total MTX exposures incl. switches)	Mono 7.5-10 mg/week; Combo + prednisone	~83% favorable response in MTX-exposed; cohort recurrence 13.6%	Mean FU ~12 months	Early evidence for steroid-sparing benefit
Aghajanzadeh et al. [32]	Iran (Rasht)	Retrospective; 2006-2013	206	56	Combo MTX 7.5-10 mg/week + steroids	71% remission in MTX group; overall recurrence 5%	Tx 2-4 months; FU 9-18 months	Step-up after steroid failure/relapse
Joseph et al. [33]	USA (NYC)	Retrospective; 2012-2013	24	7	Combo MTX 15 mg/week after steroid relapse	~80% remission with prednisone+MTX; AEs: hair loss, LFT ↑	Mean FU 15 months	No surgery needed in MTX group
Akbulut et al. [34]	Turkey	Review (1972-2010) + 4 new cases	541 (reviewed)	16 (12 L + 4 new)	Mono/Combo 7.5-15 mg/week	87.5% remission (14/16); relapse 12.5%	Tx 2-12 months; FU 2-9 months (new cases)	All 4 new cases in series: complete remission, no recurrence
Akbulut et al. [35]	Turkey	Prospective 4 case series + review	4 (+ review of 12)	4	Mono 7.5→15 mg/week (± prior therapies)	100% remission in new 4; 0% recurrence; literature 83.3% CR	FU 4-8 months	Steroid-resistant/intolerant or primary MTX; no major AEs

Overall, these findings align with growing evidence supporting MTX as the cornerstone of medical management for IGM, particularly when used as long-term monotherapy (greater than or equal to one year). The high remission rates and low recurrence rates across studies underscore its role as an effective steroid-sparing agent that minimizes the need for repeated drainage or disfiguring surgery. Furthermore, prolonged follow-up in several cohorts demonstrated sustained drug-free remission, indicating durable disease control after cessation. The observed variability in relapse, often associated with shorter MTX exposure or hormonal factors, highlights the importance of individualized treatment duration and close follow-up. Taken together, the collective data from observational studies corroborate the clinical improvement observed in our presented case, where the patient achieved marked resolution following the initiation of MTX after inadequate response to corticosteroids and I&D. These converging results emphasize MTX’s growing status as the first-line immunosuppressive agent in steroid-resistant or recurrent IGM, offering both efficacy and long-term stability with favorable safety and tolerability profiles.

## Conclusions

IGM presents significant diagnostic and clinical challenges due to its ability to mimic breast malignancy and its unpredictable, relapsing course. While corticosteroids remain a commonly used first-line therapy, prolonged treatment is often required, and recurrence or treatment-related toxicity is frequent. This case demonstrates a dramatic and sustained response to MTX after conventional therapies, including I&D and long-term steroid use, failed to achieve disease control. The favorable clinical and radiologic outcomes observed support the role of MTX as a valuable steroid-sparing option, particularly in patients with recurrent or refractory disease. Given the lack of universal guidelines, individualized management and early multidisciplinary involvement are essential to avoid unnecessary surgical morbidity and to optimize long-term outcomes in IGM.
